# Improper Report of *Schistosoma haematobium* and Associated Vesical Carcinoma in a Young Man from Iran

**DOI:** 10.18502/ijpa.v15i3.4214

**Published:** 2020

**Authors:** Alireza NOURIAN, Seyedhossein HEKMATIMOGHADDAM, Alireza SAZMAND

**Affiliations:** 1. Department of Pathobiology, Faculty of Veterinary Science, Bu-Ali Sina University, Hamedan, Iran; 2. Department of Laboratory Sciences, School of Paramedicine, Shahid Sadoughi University of Medical Sciences, Yazd, Iran

## Dear Editor-in-Chief

We read with great interest the article entitled “Vesical schistosomiasis and squamous cell carcinoma associated with schistosoma haematobium: A re-emerging neglected tropical disease in Tehran, Iran” by Haghighi et al (1), published in “Urology Case Reports”. Other than the poor English writing that catches the attention at first glance, the authors have made many egregious errors in their paper. Following are some serious issues worth to be addressed:

In the title, authors wrote the name of *Schistosoma haematobium* wrongly as “schistosoma haematobium”. According to the binomial nomenclature system, each living organism is assigned a scientific name consisting of “genus” and “species”, both of which must be written in italic, with the genus name capitalized (2). In this article the genus name in the “title” neither is capitalized nor italic.

The very first sentence of abstract is started with a species name mentioning the “species” name alone, which begun with a capital letter that is not scientifically correct. The authors also used the term “renal tube” that is a weak substitute for “renal tubule” of nephron, the functional unit of kidney, which has certainly nothing to do with *S. haematobium* infection.

In the introduction, schistosomiasis has been introduced as the most common tropical disease after malaria in the world with an unclear justification for being a “common tropical disease”. Existing in 75 to 76 countries, schistosomiasis infects more than 230 to 250 million people annually and 779 million people are at risk of infection (3). According to WHO, the death estimates due to schistosomiasis need to be re-assessed, as it varies between 24,072 and 280,000 per year globally (3, 4). However, based on the data from the WHO, 14 diseases caused by parasites, bacteria and viruses are classified as tropical diseases (5) out of which, soil-transmitted helminths are responsible for infection of more than 1.5 billion people, or 24% of the world’s population (6).

The authors used the term *“*urinary pain” which is not clear if they meant difficulty in passing urine or painful micturition. The authors could use the term “dysuria” although there are some discussions on this term too (7).

In the case report section, the authors have reported “3% eosinophilia”. The term “eosinophilia” is defined by the absolute count of eosinophils, not their percentage/proportion (8). They also used the term “pilocalix” which is incorrect form of “pyelocalyx”. The authors stated “Cystoscopy showed four tumors in the bladder and chronic granulomatous cystitis”, while the diagnosis of tumors or granulomatous reactions needs thorough microscopic examination of the tissue.

It is also mentioned that “Histopathological examination confirmed *S. haematobium* (Male and Female) were found together on the bladder wall (Fig. 1)”, however, the figure does not show the presence of both or either sexes. More importantly, none of the figures is provided with essential information including scale bar, magnification number and staining method(s). The sizes of eggs in “figure 2 (B and C)” are almost equal despite different magnifications of the micrographs. Furthermore, it seems that the terminal spines of the eggs in “figure 2 (F)” are located at the opposite pole, not at the asterisk.

It is claimed by the authors that, “During retrograde radiographic evaluations, thickening and linear calcification of the bladder and left unilateral dilation of the ureter were observed (figure 3)”. This is not correct, as thickening of the bladder wall cannot be observed using this technique. Linear calcification can be detected through plain radiography, computerized tomography (CT) scanning and ultrasonography, but not with retrograde radiography (retrograde cystography).

In “figure 2 (A)”, the authors have stated: “Squamous cell carcinoma (SCC) (superficial), transitional cell carcinoma (TCC) (in deeper layer)”. These lesions look more likely to be granulomatous reactions but not malignancy. Moreover, squamous metaplasia is by definition, different from SCC, although the first condition may be a precursor for the latter one (9). Similarly, the degeneration of transitional epithelium is quite different from TCC, and the figure does not show either SCC or TCC. Simultaneous occurrence of SCC and TCC in body is extremely rare, and must have been proved by an experienced pathologist examining high-resolution micrographs.

It should be noted that ethics approval for research is a necessity, which must have been included in the text accordingly, especially as inferred from the context, a considerable amount of tissue might have been obtained by vesical biopsy.

To conclude, it is principally accepted that individuals with relevant specialties should be involved in every research work, for instance, at least a pathologist or urologist should have been included in the authors’ team to avoid inaccuracies and deficiencies. Moreover, it is of importance that authors of scientific communications always be welcoming comments and critics to their works. Finally, in order to fulfill their mission, the scientific journals for the sake of science, must practice sound and prudent processing of articles, most importantly through proper peer-reviewing of the submitted manuscripts.
